# Risk factors of child physical abuse by parents with mixed anxiety-depressive disorder or posttraumatic stress disorder

**DOI:** 10.3325/cmj.2011.52.25

**Published:** 2011-02

**Authors:** Katija Kalebić Jakupčević, Marina Ajduković

**Affiliations:** 1 University Hospital Split, Split, Croatia; 2 Department of Social Work, Faculty of Law, University of Zagreb, Zagreb, Croatia

## Abstract

**Aim:**

To determine the risk that parents with mixed anxiety and depressive disorder (MADD) or posttraumatic stress disorder (PTSD) will physically abuse their child and evaluate the specific contribution of mental health, perceived social support, experience of childhood abuse, and attributes of family relations to the risk of child physical abuse.

**Method:**

The study conducted in 2007 included men (n = 25) and women (n = 25) with a diagnosis of MADD, men with a diagnosis of PTSD (n = 30), and a control sample of parents from the general population (n = 100, 45 men and 55 women) with children of elementary school age. General Information Questionnaire, Child Abuse Experience Inventory, Perceived Social Support Scale, and the Child Abuse Potential Inventory (CAPI) Clinical Abuse Scale were used.

**Results:**

Total results on the Clinical Abuse Scale of the CAPI indicated higher risk of child physical abuse in parents with MADD (273.3 ± 13.6) and in fathers with PTSD (333.21 ± 17.98) than in parents from the general population (79.6 ± 9.9) (F = 110.40, *P* < 0.001; t_PTSD,MADD_ = 13.73, *P* < 0.001). A hierarchical regression analysis showed that the greatest predictors in the multivariate model were mental health difficulties, poorer economic status, poor social support, and physical and verbal aggression in partner conflicts.

**Conclusion:**

Parents with MADD and PTSD exhibit high risk of child abuse. Since parents with PTSD have significantly higher risk of child abuse than parents with MADD, further large-sample research is needed to clarify the relationship between PTSD intensity and the risk of child abuse.

The World Health Organization lists family violence as one of the most important contemporary public health issues. It defines physical abuse of children as “any intentional use of physical action against a child that causes or is likely to cause harm to the child’s health, survival, development or dignity, including beating, kicking, shaking, biting, strangulation, scalding, burning, deliberate poisoning and suffocation, or failure to prevent physical injury (or suffering)” (1).

Contemporary theoretical models explain child abuse in the family as a complex phenomenon caused by an interaction of multiple factors (2,3) at different ecological levels – individual, relational, community, and societal. In this study, the focus is on parent-related individual and relationship factors. The individual risk factors include circumstances in the parent’s personal history that can increase the risks for child abuse. Previous research has focused on the history of maltreatment in parents’ childhood, mental health problems, lack of self-control when upset or angry, misuse of alcohol or drugs, social isolation, poor parenting skills, positive attitude toward the use of physical punishment as a means for discipline, and financial difficulties. The most important relationship risk factors include mental health problems of family members, marriage or intimate relationship difficulties, partner violence in the family, lack of support network in stressful or difficult life situations, and lack of support in child rearing from family members (3).

Although international research demonstrated that psychiatric disorders were an important risk factor for child maltreatment (4-8), no research focusing on this topic has been carried out in Croatia so far. International research indicated that the most frequently analyzed groups of disorders in child-abusing parents were anxiety disorders, depression, antisocial behaviors, personality disorders, and dissociative disorders. However, most of the previous studies have neglected several important methodological concerns. First, the parents who are usually included in these studies are the mothers, whereas the study of the fathers’ mental health problems associated with child abuse has received much less attention. Second, posttraumatic stress disorder (PTSD) is rarely included in research as a predictor or risk factor for abuse – it is more often studied as a consequence of exposure to family violence (9-11). Also, while there are studies on mental health difficulties of parents who have been registered for violence against children, clinical populations of parents have been much less studied.

The focus of this research is the risk assessment of child abuse by parents of both sexes who have not been registered as perpetrators of violence against children, and who have a diagnosis of a psychiatric disorder – PTSD or mixed anxiety and depression disorder (MADD). Starting from ecosystem and interactive models of violence against children in the family, mental disorders of the parents were considered in interaction with their socio-demographic characteristics and personal histories (such as childhood abuse), as well as current family dynamics (such as social support and quality of partner relations). The aims of this study were:

1. To compare the risk of physical child abuse, perceived social support, experience of childhood abuse, and some characteristics of family relations between parents with MADD, parents with PTSD, and parents from the general population.

2. To test the contribution of mental health, perceived social support, the experience of childhood abuse, and some features of family relations to the risk of physical child abuse by parents with MADD or PTSD and by parents from the general population.

## Participants and methods

### Participants

The study was carried out in 2007 on a convenience sample of two clinical groups of patients: 1) men (n = 25) and women (n = 25) with a psychiatric diagnosis of mixed anxiety and depression disorder (International Classification of Diseases, 10th revision [ICD-10] code F 41.2) (12) and 2) men (n = 30) with a psychiatric diagnosis of PTSD (ICD-10 code F 43.1) due to war trauma.

These participants met the following inclusion criteria: had no other psychiatric disorders in medical history, were outpatients in a psychiatric clinic without previous hospitalization, their psychiatric treatment was longer than 5 years, had an elementary school-aged child, and were not single parents. It was initially planned to include mothers with PTSD as well, but it was impossible to find a sufficient number of women according to the predefined sampling criteria that would allow the planned statistical analyses. The data on the imbalance between the number of men and women with PTSD are consistent with previous data on persons with PTSD in Croatia. For example, in a study on the work capacity and social functioning of persons with PTSD, only 15 women were included in a sample of 160 persons (13).

The comparison group consisted of 100 parents (45 fathers and 55 mothers) who met the following criteria: had elementary school-aged children, reported that they had never been treated by a mental health professional, and were not single parents.

The data on parents with MADD and PTSD were collected through individual interviews carried out by psychologists working in a mental health clinic. They were interviewed during their regular check-ups in a psychiatric outpatient clinic. The participants were informed of the purpose of the study and their participation was voluntary. The study was approved by the Clinical Hospital Split and by the Ethics Committee of the Faculty of Humanities and Social Sciences, University of Zagreb.

The data on the subsample from the general population were collected by psychologists in a group setting in various schools in the wider Split area. Participation was voluntary and anonymous, and was carried out in line with the Code of Ethics of Psychological Activities (14).

### Instruments

Four questionnaires were used: General Information Questionnaire, which was used to collect socio-demographic data and data on family relations, the Child Abuse Experience Inventory, Perceived Social Support Scale, and the Child Abuse Potential Inventory-Clinical Abuse Scale.

The General Information Questionnaire was constructed for this study to collect the data on the self-reported socio-demographic characteristics of the parents: age, sex, marital status, educational status, employment status, family financial status (including social benefits), and the number of children. The questionnaire also included questions on family relations. The following aspects of family relationship were measured: satisfaction with partner relations, partner participation in child rearing, frequency of verbal and physical partner conflicts, satisfaction with support received from extended family and friends. These items were assessed on 1 to 5 point scales, where 5 indicated a more favorable situation. The frequency of alcohol consumption was assessed by one item on the scale: 1 (never), 2 (rarely), 3 (several times during month), and 4 (several times during week). One item asked if the person had ever been treated for mental health problems and was used as exclusion criterion for the comparison group.

Child Abuse Experience Inventory was developed and validated in Croatia in 2001 (15). It was developed according to the Comprehensive Child Maltreatment Scales for Adults (16). The scale consists of 19 items that describe the experience of emotional abuse (7 items), physical abuse (4 items), neglect (5 items), and exposure to violence between parents (3 items) during childhood. The participants are asked to indicate on a four-point scale (0 – never, 1 – sometimes, 2 – often, 3 – almost always) the frequency of experienced abuse/neglect separately from mother and father (ie, “Did your mother/father punish you without reasons?;” “Did your mother/father beat you?;” “Did your mother/father mock you?”). A higher score indicates more intense and more frequent experience of abuse. In this study, the coefficient of internal reliability of the scale (Cronbach α) was = 0.81. The high internal reliability indicates unidimensionality of the inventory and therefore in this study the total score was used. The total score was also used in previous studies, which showed that manifestation of only one type of abuse in childhood is an exception and that children are typically exposed to several types of abuse (17,18).

Perceived Social Support Scale (19) provided a general assessment of the degree of support a person expects from significant others in the social network. This scale is an adaptation of the scale by Simons et al (20). It consists of 11 items describing the availability of various forms of social support in a person's environment. The participants rated a 5-point scale the degree to which the items applied to them. The total score was computed as a sum of responses on the 11 items, with a higher score indicating better perceived availability of social support. The internal consistency coefficient (Cronbach α) of the scale in this study was 0.80.

Child Abuse Potential Inventory (CAPI) (21) was designed to assess the parents’ risk of physically abusing their children. The CAPI is a widely used self-report measure consisting of 160 statements with which the respondents must either agree or disagree. Seventy-seven of the items are variably weighted, contributing to the overall Clinical Abuse Scale scores, whereas the remaining items include the questions used in the three validity scales. In terms of predictive validity, the CAPI has been found to be an accurate predictor, correctly identifying 81.4% of confirmed child abusers and 99% of the parents from the control group. Reliability coefficients have been found to range from 0.92 to 0.95 for the whole scale (21,22).

In the current study, only the 77-item Clinical Abuse Scale was administered, to reduce the burden for the participants. Previous research has shown that the reliability of the Clinical Abuse Scale in the Croatian version of the questionnaire is high (Cronbach α = 0.91) (23), and that it is efficient in discriminating abusing/neglecting parents from parents in the general population. The reliability of the scale (Cronbach α) in the present study was 0.97. The result on the CAPI Clinical Abuse Scale is formed as a weighted linear combination of the 77 items of the clinical scale. The theoretical range of results is 0-486. Higher scores indicate a greater potential for physical child abuse. The cut-off values, above which the results can be interpreted as the presence of physical abuse or a high risk of physically abusing the child, is 215, which is the top 5% of the participants in the control sample of the parents who do not abuse their children (21).

The Clinical Abuse Scale includes 6 subscales based on a factor analysis of the items. The first three factors describe psychological difficulties of the respondents, while the three remaining factors relate to problems that the respondents experience in social relationships:

1. Distress (36 items). The factor is defined by manifest variables of frustration, sadness, loneliness, feeling low, worry, fear, lack of control over the environment, confusion, being upset, tension, worthlessness, rejection, lack of understanding, and anger. The theoretical range of the results is 0 to 261.

***2.*** Rigidity (14 items). The factor concerns the rigidity of the parent's attitudes toward the child's appearance, behavior, and upbringing, including the belief that children need strict rules. The theoretical range of the results is 0 to 64.

3. Unhappiness (11 items). The factor concerns general dissatisfaction with life, as well as specific components of dissatisfaction related to difficulties in close interpersonal relationships. The theoretical range of the subscale is 0 to 69.

4. Problems with Child and Self (6 items). The factor measures negative perception of the child's and the parent's physical characteristics. It includes the perception of the child as being slow, having special difficulties, being often in trouble, and being “naughty.” Besides that, the factor includes physical and health problems of the parents themselves. The theoretical range of the subscale is 0 to 30.

5. Problems with Family (4 items). The factor includes various difficulties in family relationships of the parents. The items include conflicts in the family, disagreement among the parents, and having a large number of family problems. The theoretical range of the subscale is 0 to 38.

6. Problems with Others (6 items). The factor indicates general difficulties in social relationships that are a source of distress, unhappiness, pain, and disappointment of the respondent. The theoretical range of the results is 0 to 24.

### Statistical analyses

The distributions of the results are presented as means ± standard deviations. Analyses of variance (ANOVA) were used to test differences among groups. ANOVA was conducted with mental health status as independent variable (patient with PTSD, patient with MADD, and parents from the general population). Post-hoc Sheffe tests were used to test for differences between pairs of groups in ANOVA.

Since the groups were not balanced by sex (persons with PTSD were male only), two-way analysis was used to test for the effects of sex by clinical and the comparison group on the risk of physical abuse of children, perceived social support, the experience of childhood abuse, and the characteristics of family relations. The effect of sex was not significant for all of these variables, so further analyses were carried out on groups defined by diagnoses.

Hierarchical multiple regression analysis was conducted to predict Clinical Abuse Scale scores. The regression proceeded with potential background/demographic covariates in the first block, the experience of childhood abuse was entered in the second block, followed by the mental health of the parent, characteristics of family relations, and perceived social support in the final, fifth block.

The level of statistical significance was set at *P* < 0.050. Statistical analyses were performed with the statistical package STATISTICA 7 (StatSoft, Tulsa, OK, USA).

## Results

### Socio-demographic characteristics

The mean age of the participants with PTSD was 44.9 ± 5.69 years. They assessed their financial status as average (2.7 ± 0.5 out of max 5); 73.3% had never requested material aid from a center for social welfare, and 33.33% were employed. Fathers with PTSD had predominantly finished high school (73.3%), followed by elementary school (20%), and college/university (0.7%). The majority had one or two children (76.66%).

The mean age of participants with MADD was 40.5 ± 6.2 years. Their self-assessed financial status was between below-average and average (2.5 ± 0.6 out of max 5), 47.9% were employed, and 74% had never requested material aid from a center for social welfare. The majority of parents with MADD had finished high school (76%), followed by elementary school (14%), and college/university (10%). The vast majority had one or two children (90%).

The mean age of parents from the general population was 37.6 ± 6.2 years. Their self-assessed financial status was average (3.0 ± 0.4), 74.7% were employed, and 96% had never requested material aid from a center for social welfare. The majority had finished high school (60%), followed by college/university (36%). Only one parent from that group had only elementary education (1%). The majority had one or two children (82%).

Male participants with MADD and those with PTSD did not differ in the number of children (*t* = -0.75, *P* = 0.452), educational status (*t* = -0.56, *P* = 0.571), and self-assessed financial status (*t* = -1.32, *P* = 0.190). Women were significantly younger than men (*t* = 2.19, *P* = 0.030), but this difference was not relevant for the interpretation of further results in this study.

Compared with the clinical subsamples, parents from the general population were better educated (*t* = 6.19, *P* < 0.001), had better self-assessed financial status (*t* = 5.98, *P* < 0.001), and were younger (*t* = -4.86, *P* < 0.001), but they did not differ in the number of children (*t* = -1.73, *P* = 0.084).

Regarding frequency of alcohol consumption, the significant difference was found between participants with PTSD (2.5 ± 0.9 out of max. 4) on the one hand, and participants with MADD (1.8 ± 0.7 out of max. 4) and participants from the general population (1.8 ± 0.8 out of max. 4) on the other (F = 9.19, *P* < 0.001).

### Risk of physical abuse among groups of parents

The first aim of this study was to compare the risk of physical child abuse by parents with MADD, parents with PTSD, and parents from the general population (Table 1).

**Table 1:** Scores on the Clinical Abuse Scale (CAS) and its individual subscales in parents from the general population (COMP), parents with anxiety and depression disorder (MADD), and parents with posttraumatic stress disorder (PTSD).

	Patients (mean ± standard deviation)					
	control (n = 100)	with MADD (n = 50)	with PTSD (n = 30)	F-ratio	*P*	Sheffe post hoc*
Distress	46.7 ± 51.1	178.4 ± 75.6	207.5 ± 66.1	103.5*	<0.001	COMP-MADD COMP-PTSD
Rigidity	17.2 ± 13.4	28.4 ± 12.1	40.0 ± 13.7	35.37*	<0.001	COMP-MADD COMP-PTSD MADD-PTSD
Unhappiness	10.7 ± 9.9	40.9 ± 18.4	45.0 ± 19.2	97.3*	<0.001	COMP-MADD COMP-PTSD
Problems with child and self	2.5 ± 4.76	2.0 ± 4.5	5.46 ± 7.9	4.4*	0.014	COMP-PTSD MADD-PTSD
Problems with family	3.6 ± 6.2	11.3 ± 11.2	12.9 ± 12.0 01	19.13*	<0.001	COMP-MADD COMP-PTSD
Problems with others	5.4 ± 6.2	15.89 ± 7.2	16.9 ± 7.5	55.16*	<0.001	COMP-MADD COMP-PTSD
Total score on CAS	79.6 ± 9.9	273.3 ± 13.6	333.2 ± 18.0	110.4*	<0.001	COMP-MADD COMP-PTSD MADD-PTSD

*P < 0.05, post-hoc tests.

Compared with parents from the general population, parents with MADD had a significantly higher risk of child abuse on 5 of the 6 subscales (with the exception of “Problems with Child and Self”), while fathers with PTSD had higher risk of child abuse on all of the subscales (Table 1).

The total results on the Clinical Abuse Scale suggest a high risk of physical child abuse by parents with MADD (73.3 ± 13.6) and fathers with PTSD (333.2 ± 18.0), compared with parents from the general population (79.6 ± 9.9), whose total score was below the cut-off value of 215.

Fathers with PTSD reported more rigidity (40.0 ± 13.7) and more problems with child and self (5.5 ± 7.9) than the group with MADD (rigidity 28.4 ± 12.1; problems 2.0 ± 4.50). Fathers with PTSD had a significantly higher potential for child abuse than parents with MADD, which suggests that their risk of child abuse is especially high (*t* = 13.73, *P* < 0.001).

All subscales of the Clinical Abuse Scale were significantly correlated, with the total score ranging from 0.98 for distress to 0.43 problems with others. Therefore, the total score was used as a criterion variable in further analyses.

### Personal experience of childhood abuse and some characteristics of family and social relations

Parents from the general population and parents with MADD or PTSD differed in their own experience of childhood abuse and in some characteristics of family and social relations (Table 2). Parents with MADD or PTSD reported more experiences of childhood abuse (F = 14.46, *P* < 0.001), as well as more exposure to parental conflicts in childhood (F = 5.71, *P* < 0.001) than parents from the general population. Parents with MADD or PTSD did not differ between themselves in these variables. Parents from the general population also reported better perceived social support (F = 27.55, *P* < 0.001) and support in child rearing from friends (F = 7.07, *P* < 0.001) than parents with MADD or PTSD. The two clinical groups had comparable results in these variables. Parents with PTSD reported that their partners participated more in child rearing than themselves compared with parents from the general population and parents with MADD (F = 7.72, *P* < 0.001), whereas parents with MADD reported less family support in child rearing than parents from the general population (F = 3.72, *P* = 0.020).

**Table 2:** Scores on the scale of perceived social support and scale for the assessment of childhood abuse in parents from the general population (COMP), parents with anxiety and depression disorder (MADD), and parents with posttraumatic stress disorder (PTSD).

	Patients (mean ± standard deviation)					
	controls (n = 100)	with MADD (n = 50)	with PTSD (n = 30)	F-ratio	*P*-value	Sheffe post hoc*
Experience of childhood abuse	28.7 ± 6.4	36.5 ± 11.6	33.9 ± 7.2	14.46*	<0.001	COMP-MADD COMP-PTSD
Parental conflicts in childhood	4.4 ± 1.5	5.1 ± 2.0	5.4 ± 1.9	5.71*	<0.001	COMP-MADD COMP-PTSD
Perceived social support	42.6 ± 7.5	36.3 ± 5.3	33.25 ± 5.8	27.55*	<0.001	COMP-MADD COMP-PTSD
Participation of partner in child care	2.9 ± 0.6	3.0 ± 1.0	3.5 ± 0.6	7.72*	<0.001	COMP-PTSD MADD-PTSD
Family support in child rearing	3.7 ± 1.1	3.3 ± 1.1	3.4 ± 0.8	3.72*	0.020	COMP-MADD
Friends' support in child rearing	3.6 ± 0.8	3.1 ± 0.9	3.0 ± 0.8	7.07*	<0.001	COMP-MADD COMP-PTSD
Assessment of partner relationship	4.3 ± 0.7	3.2 ± 1.0	3.2 ± 0.8	36.15*	<0.001	COMP-MADD COMP-PTSD
Frequency of conflicts with partner	2.4 ± 1.1	3.3 ± 1.0	3.5 ± 1.2	16.06*	<0.001	COMP MADD COMP-PTSD
Conflicts – physical aggression	1.0 ± 0.2	1.4 ± .9	1.7 ± 1.0	15.28*	<0.001	COMP-MADD COMP-PTSD
Conflicts – insults	1.4 ± 0.79	2.8 ± 1.01	2.9 ± 1.26	52.83*	<0.001	COMP MADD COMP-PTSD

*P < 0.05, post hoc tests.

Parents with PTSD or MADD rated their relationships with partners similarly poorer than parents from the general population (F = 36.15, *P* < 0.001). The clinical groups also reported more frequent conflicts with partners (F = 16.06, *P* < 0.001), physical aggression (F = 15.28, *P* < 0.001), and insults (F = 52.83, *P* < 0.001) than parents from the general population (Table 2).

### Predictors of risk of physical abuse of children

The mental health status of the participants was defined as bivariant variable – parents with MADD or PTSD vs parents from the general population. Before the hierarchical regression analysis, the univariant analyses for the 15 variables related to different sociodemographic characteristics, alcohol consumption, experience of childhood abuse, characteristics of family relations, and perceived social support were done with mental health status as the bivariant variable. Alcohol consumption (*P* = 0.132) and the number of children (*P* = 0.243) were not introduced in the predictor models because the bivariant analyses showed that they did not differ on the criterion variable.

The predictor variables were introduced in blocks of variables. The first block included sociodemographic variables; the second block included the experience of childhood abuse; the third block included mental health of the parent; the fourth block included characteristics of family relations; and the fifth block included perceived social support (Table 3).

**Table 3:** Final hierarchal multiple regression predicting Clinical Abuse Scale scores in all study participants (n = 180)

Predictors	R	R^2^	ΔR^2^	F (df)	β	β in final step
First step:	0.63	0.40		19.96 (4.116)		
age					0.19*	
sex						
educational status					-0.35*	
economic status					-0.32*	-0.16^†^
Second step:	0.70	0.49	0.09	22.84 (5.115)		
experience of childhood abuse					0.31*	
Third step:	0.79	0.64	0.14	32.77 (6.114)		
mental health status					0.50*	0.24^†^
Fourth step:	0.86	0.74	0.10	23.52 (13.107)		
participation of partner in child care						
family support						
friends' support					-0.22*	
relationship with partner						
frequency of conflicts						
conflicts – physical aggression						0.12^†^
conflicts – insults					0.20*	0.14^†^
Fifth step:	0.88	0.78	0.04	27.46 (14.106)		
perceived social support						-0.34^†^

*P < 0.05, post hoc tests.

Introducing sociodemographic variables into the model explained 40% of the variance of the risk of physical abuse of children, with age, educational status, and financial status being significant in the first step. After introducing other blocks of variables in the subsequent four steps, only the financial status remained an independent predictor (β = -0.16). In the second step, the experience of childhood abuse was introduced, which increased the proportion of explained variance by 9%, however, in the subsequent steps, the contribution of this variable was shown to be not significant. In the third step, the variable of mental health difficulties was introduced, which increased the proportion of explained variance by 14%. This variable remained an independent predictor in the final step (β = 0.24). In the fourth step, the characteristics of the family dynamics explained other 10% of the variance, however, only partner physical conflicts (β = 0.12) and insults were kept as independent predictors (β = 0.14) in the final step. In the fifth step, the variable of perceived social support was introduced, which increased the proportion of explained variance by 4%, so that the total proportion of explained variance was now 77%. The independent contribution of this variable was significant (β = -0.34).

## Discussion

Based on cut-off values for evaluating the risk of child abuse (top 5% of results on the CAPI Clinical Abuse Scale) (21), our study demonstrated that parents with MADD and fathers with PTSD exhibited a great risk of child abuse. These results are in line not only with previous clinical, but also with epidemiological research. A representative community sample of 8548 respondents in the Ontario Mental Health Supplement Study were interviewed about their parents’ psychiatric history and completed a self-report measure of childhood physical and sexual abuse (4). Respondents with a parental history of depression, mania, or schizophrenia had two to three times higher rate of abuse. There was no significant difference between genders in parental psychiatric disorder or childhood physical or sexual abuse. Respondents who had both parents with a psychiatric disorder had had the greatest exposure to childhood abuse, followed by those who only had the mother with a psychiatric disorder, and finally by those who only had the father with a psychiatric disorder.

The present study revealed that fathers with PTSD had a higher risk of abusing their child than parents with MADD. Also, the relationship of parents with mental health difficulties with their partners was also worse and the frequency of conflicts accompanied by physical aggression and insults was significantly greater than in parents from the general population. The only difference found between the two clinical groups was in partner participation in child rearing, which was significantly larger in parents with PTSD than in parents with MADD. The high risk of child abuse due to mental illness of the parent is comparable with previous research (4,6).

A greater risk of child abuse in parents with PTSD than in parents with MADD can be linked with the finding that trauma and combat PTSD can cause poor attachment and frequent aggressive behaviors toward family members (24). A study on professional soldiers indicated that the partner abuse was an important predictor of child abuse (25-27). Milner and Dopke (28) found that fathers with PTSD were at a high risk to physically abuse their children, which may be associated with their lowered capacities of problem-solving, decreased tolerance, and difficulties in impulse control. They also had fewer interactions with their children, scarcer mutual communication, and less consistency toward children than parents who are not violent (28).

A Croatian study demonstrated that more intense PTSD was associated with externalized symptoms such as acting out, hostility and mistrust, while less severe PTSD was associated with mostly depressive symptoms (29). Another study showed that Croatian war veterans with PTSD who had been psychiatrically assessed as incapable to work due to their intensive symptoms expressed more dysfunctions in the parental role, participation in housework, and in general social functioning than patients with less pronounced symptoms (13). Even the 39.4% of PTSD patients who were assessed as capable to work reported having difficulties in the parental role.

Despite these findings, caution must be exercised in interpreting the link between traumatic events or the diagnosis of PTSD and the risk of child abuse: just because a person has experienced a traumatic event or has PTSD does not mean that they will exhibit violent behaviors. There are many factors that contribute to aggressive behavior, and extensive further research is needed to identify the specific risk factors for child abuse among such people, including the role of intensity of PTSD symptoms. The need for further research in this direction is highlighted by reports that children of war veterans with PTSD are not only exposed to higher risk of abuse, but that their development is generally more endangered. For example, war veterans diagnosed with PTSD from Bosnia and Herzegovina reported that their children exhibited a higher prevalence of developmental (ie, speech disorder), behavioral (leaving schools, aggressive behavior), and emotional problems (depressed mood) than children whose fathers had not been diagnosed with the disorder (30).

In this study, parents with MADD and PTSD reported the experience of childhood abuse, as well as having been exposed to their parents’ violence more frequently than parents from the general population. Their high risk of abusing their children is consistent with previous international and Croatian research. For example, a study on Croatian college students has shown that more frequent experiences of corporal punishment by parents, and abuse and violence between the parents during the childhood are related to the higher risk that individuals will abuse their own children (2). Witnessing violence between the parents and having been abused in childhood are predictors of violence in adulthood, a tendency toward social isolation, and a feeling of a lack of predictability in partner relations (31). A study on pre-military variables found a positive correlation between the severity of combat-related PTSD and a history of physical abuse (32).

Parents with PTSD or MADD perceived significantly less support from friends in child rearing than parents from the general population. It is well established that social isolation and loneliness are important risk factors for child abuse and that it is important to strengthen the patients' support networks. In the literature on combat PTSD, the posttraumatic environment is often highlighted as one of the determinants of successful adjustment (33). Of these variables, the most frequent one is social support, usually defined as the person’s assessment on how supportive their social environment is (33). The principal cause of problems in social interactions of persons with PTSD is that, being unable to modulate arousal, their volatile and “inappropriate” reactions even to minor stimuli can scare the persons in their social environment, who fail to see reasons for such rage, anxiety, fear, and other emotional reactions (32).

If one takes into account that parents who have mental health difficulties, aside from more frequent exposure to childhood violence, also receive less social support, maintain less satisfying partner relationships, and have poorer financial status, it is obvious how these parents accumulate a number of risk factors for child abuse. This population of parents needs special clinical attention, as well as more focus in research that would establish the paths that lead to violent behavior toward children and other family members.

Our findings can be used to formulate guidelines for the organization of counseling/therapeutic work with people suffering from MADD or PTSD, with the goal of preventing violence against children. The parents should be taught to build positive interactions with the child, recognize and fulfill the child's needs, and develop the capacity for containing their anger and feelings of frustration in front of the child and in other close relations (27).

In the families in which one of the partners has mental health difficulties, the other partner and the children may need additional psychosocial support. Such services could be provided through psychoeducation or through family counseling programs. This research confirmed that the poor quality of partner relations significantly contributed to the risk of physical abuse of children. The support to women victims of partner violence in fulfilling their parental role is vital, so that they can be a psychological resource to their children – education on the detrimental effects of witnessing the father's violence against the mother, as well as training on specific parenting skills is needed (23).

This research may contribute to implementation of the Program for Increasing the Quality of Life of Families of Fallen Croatian Veterans, Croatian Military War Disabled, and Croatian Veterans With PTSD (*www.mobms.hr*). This document draws attention to the phenomenon of “secondary traumatization” in 30% of the families of 7746 veterans with the status of military war disabled persons due to PTSD. It also highlights the need for research on the long-term effects of war traumatic experiences, including the effects on family members, in order to provide focused assistance and support.

This study has several limitations. First, due to the small sample, our results may not be generalizable to the wide clinical population. Second, the data on our main criterion variable, risk of the physical child abuse, were collected by the self-report questionnaire. It is possible that participants over-reported or under-reported certain risk factors. However, this limitation is present in all studies that use self-report questionnaires as the method of data collection. The third limitation is related to the characteristics of the group of parents with PTSD. This group comprised only male participants because the number of mothers with PTSD was extremely small not only in the clinical population of the psychiatric clinic where the study was done but also in other psychiatric clinics in Croatia (13). Finally, at least one study (29) found that more severe PTSD symptoms were associated with externalized symptoms, ie, aggression and acting-out behaviors and less severe PTSD was associated with mostly depressive symptoms, but in our study the participants with PTSD were treated as one group because of the small subsample size. In light of such findings, further research of the association of PTSD severity with the risk of child abuse is needed.

In conclusion, this study confirmed that parents’ mental health difficulties were a risk factor for child abuse. Both, parents with PTSD and those with MADD exhibit a high risk of child abuse. Aside from mental health difficulties, perceived poor social support, poor self-assessed financial status, and more frequent partner conflicts, including insults and physical aggression, are most important in predicting the risk of physical abuse of children.

Since parents with PTSD are at significantly higher risk of child abuse than parents with MADD, further research is needed to establish the risk of child abuse relative to the intensity of PTSD. Given that parents with mental health problems reported a higher prevalence of history of abuse in childhood, poor social support, and conflicts in the current family relationships, research models that would allow the application of structural equation are needed. Such research designs would provide more reliable data on the specific ways how PTSD predicts child abuse compared with other risk factors.
